# Aerosol Transmission of SARS-CoV-2: Physical Principles and Implications

**DOI:** 10.3389/fpubh.2020.590041

**Published:** 2020-11-23

**Authors:** Michael C. Jarvis

**Affiliations:** School of Chemistry, Glasgow University, Glasgow, United Kingdom

**Keywords:** evaporation, wind, turbulence, ventilation, ultraviolet, mask

## Abstract

Evidence has emerged that SARS-CoV-2, the coronavirus that causes COVID-19, can be transmitted airborne in aerosol particles as well as in larger droplets or by surface deposits. This minireview outlines the underlying aerosol science, making links to aerosol research in other disciplines. SARS-CoV-2 is emitted in aerosol form during normal breathing by both asymptomatic and symptomatic people, remaining viable with a half-life of up to about an hour during which air movement can carry it considerable distances, although it simultaneously disperses. The proportion of the droplet size distribution within the aerosol range depends on the sites of origin within the respiratory tract and on whether the distribution is presented on a number or volume basis. Evaporation and fragmentation reduce the size of the droplets, whereas coalescence increases the mean droplet size. Aerosol particles containing SARS-CoV-2 can also coalesce with pollution particulates, and infection rates correlate with pollution. The operation of ventilation systems in public buildings and transportation can create infection hazards via aerosols, but provides opportunities for reducing the risk of transmission in ways as simple as switching from recirculated to outside air. There are also opportunities to inactivate SARS-CoV-2 in aerosol form with sunlight or UV lamps. The efficiency of masks for blocking aerosol transmission depends strongly on how well they fit. Research areas that urgently need further experimentation include the basis for variation in droplet size distribution and viral load, including droplets emitted by “superspreader” individuals; the evolution of droplet sizes after emission, their interaction with pollutant aerosols and their dispersal by turbulence, which gives a different basis for social distancing.

## Introduction

Liquid or solid particles <5–10 μm in diameter are classed as aerosol-sized and remain suspended in the air over times of seconds to hours (1), whereas particles or droplets above this threshold diameter settle quickly out of still air onto surfaces. Contrary to initial guidance (2), there is growing evidence that airborne transport in aerosol particles is significant in the spread of SARS-CoV-2, in addition to infection via larger droplets from coughing or sneezing via and surface deposits (fomites) (3). It was initially questioned whether SARS-CoV-2 was viable in aerosols, and thus whether the presence of infective virus could be inferred from viral RNA (1, 4). A number of studies have now shown that the virus does remain viable in aerosols with a half-life of about an hour indoors (5–8). Because aerosol transmission does not require coughing but is possible through normal breathing (9, 10), asymptomatic individuals, known to be carriers of COVID-19 infection (11), can infect others by this route (12–14). The quantitative importance of aerosol transmission relative to transmission by other routes is still under debate (15–17) and may vary between environments, but the precautionary principle demands that measures to block this transmission route should be vigorously adopted (18). The behavior of aerosols in indoor and outdoor environments differs in its physical basis from the behavior of larger droplets (1), and different, additional containment measures are therefore needed (19, 20).

This minireview covers mechanisms of aerosol emission, evolution and transport, together with some implications for SARS-CoV-2 transmission in non-clinical public buildings and transportation. Transmission in hospital settings, including generation of aerosols during clinical procedures involving COVID-19 patients, and fecal bioaerosols from toilets are excluded, having been reviewed elsewhere (21–24).

The literature survey was based on Web of Knowledge searches, including “snowball” searching, up to August 2020. Preprints that have not yet been peer reviewed are excluded.

## Physical Principles

### Aerosol Generation

During coughing and sneezing, liquid droplets with a wide diameter range from sub-μm to >100 μm are atomised from saliva and from fluids further down the respiratory tract (25, 26). It is now recognized that normal breathing and speech atomise droplets also (9, 27–29). Half a minute of speech releases a liquid volume comparable to a cough (10). The volume of droplets emitted during speech depends on loudness (30, 31) and may be greater during singing (1, 30). The breath emission rate is considerably increased during physical exercise (32).

The formation of aerosols and larger droplets within the respiratory tract, involving disruption of mucus layers by abrupt airflows, has been reviewed (10, 25, 33). The details differ between the lower respiratory tract, which is the principal atomisation site during normal breathing (25, 34), and the laryngeal and oral/nasal regions where further droplets are created during speech, coughing and sneezing (35–37). Each site has a characteristic droplet size range (10, 36). Aerosol-sized (~1–3 μm) droplets are produced in the lower respiratory tract and the laryngeal region (36), and any larger bronchial droplets may be redeposited before exhalation (38). Large droplets up to 500 μm come from the oral and nasal cavities (35, 36). When generated by speech these vary with loudness (30, 36) and articulation (30, 31, 35, 39).

**Figure 1:**
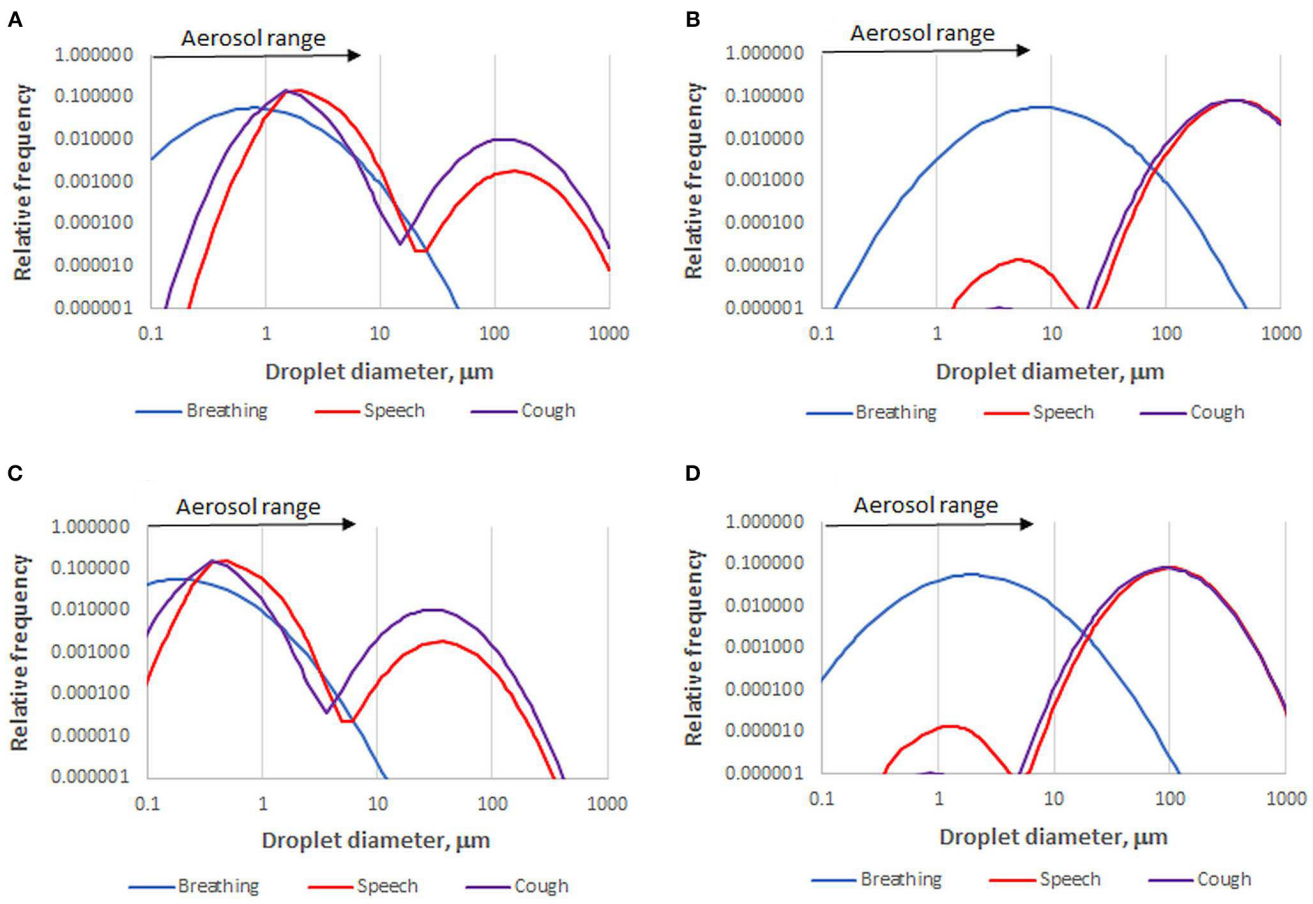
Number-based **(A)** and volume-weighted **(B)** diameter distributions for one data set of droplets emitted by healthy subjects during breathing, speech, and coughing [Sneezing gives a distribution similar to coughing but with more >100 μm droplets (26, 43)]. Data recalculated from (36). A skew to larger diameter is inherent in volume weighting. For example, speech droplets within the aerosol size range in this data set comprise 96% of the total number but only 0.01% of the total volume. The corresponding fully dried droplet diameter distributions **(C,D)** are based on the assumption that the volume of dried solutes is 1.4% of the original droplet volume.

Overall droplet size distributions for speech, coughing and sneezing depend on the relative contributions of each site of origin. However, caution is needed. Published size distributions vary greatly due to differing instrumental sensitivity, especially for large droplets, and wide variation between individuals (27, 40, 41). Droplet size distributions can be continuous (26), bimodal (42, 43), or trimodal (27, 36). They are often presented on a number basis (36), which is more robust than a volume-weighted basis when comparing measurements by methods that vary in their upper diameter limit. Figure 1 shows that a broad or bimodal size distribution is very different when presented on number and volume-weighted bases. It might be suggested that volume-weighted distributions (26) give a better indication of how the virus is distributed across the spectrum of droplet sizes, but that assumes the largest droplets are adequately measured and the viral concentration is constant whatever the droplet size and origin, which it is not (44): the disease progresses downward from the nasal region (45), therefore the origin and droplet size range of exhaled virus changes with disease progression. Other influences include effects of infection on airway surfaces (34, 40, 46, 47) age (45, 48) and conceivably viral genotype (49).

It would be useful to know whether “superspreader” events (40, 50) involve specific droplet size distributions, large emitted volumes, high viral load or a combination of these factors. Very wide variation between subjects (x10^5^ or more) has been observed for droplet volumes emitted during breathing (51) and for viral load (25, 27–29, 33, 36, 40, 46, 52–54).

### Drying of Aerosol Droplets

Droplets are exhaled in water-saturated air and dry quickly to what in the medical literature is called a *droplet nucleus* (27). Particularly for an initial diameter of some tens of μm, drying can convert droplets large enough to settle out of the air into aerosol-sized particles that remain suspended (55). Whether droplets dry on the relevant timescale of seconds can depend on either kinetics or thermodynamics. The kinetic effect is the dominant factor for large droplets, whereas the thermodynamic effect [i.e., the equilibrium water content of the droplet in contact with the ambient air], is dominant for aerosol-sized droplets (55).

Aerosol evaporation kinetics are well-understood in combustion science. In the size range above 1 μm the evaporation rate depends on the square of the diameter (D^2^ rule) (56) and on temperature (56). For water droplets it also depends on absolute humidity (57) and turbulent flow (58). The drying conditions are not constant, because the temperature and humidity of the breath or cough plume decrease as it mixes with ambient air (32). In dry air 1 μm droplets dry in milliseconds, 10 μm droplets in tenths of second and 100 μm droplets in about 1 min (10). For comparison, 10 μm droplets take several minutes to settle to the floor from 1.5 m in still air (57), whereas 100 μm droplets settle in about 5 s (10). The settling has been visualized by laser sheet imaging (28, 43).

After 1 s, droplets of aerosol size, <5–10 μm, have had time to dry to equilibrium. Their equilibrium moisture content depends on the relative humidity, their salt content which determines the water activity, and for smaller particles, their size through the Kelvin effect (55). Exhaled droplets become completely dry at 50–70% relative humidity and their equilibrium water content increases, roughly exponentially, at higher relative humidity levels (42, 59). Droplet drying, along with settling and entrainment in cough airflows, has been modeled by computational fluid dynamics (55, 60, 61) to make important predictions about virus transmission in confined settings. In some of these studies (60, 61), an unrealistically high salt content was assumed [100 g/L NaCl, compared with <10 g/L salts in saliva (62)] so that the dry diameter and settling rate were considerably overestimated. A droplet with 1.4% solids content, mainly organic (55), is reduced in diameter by a factor of about four on complete drying (Figure 1).

Drying equilibria for aerosol droplets are also modeled in atmospheric science, where the term *droplet nucleus* is not used but the concept is well-understood, and the effect of salt composition is modeled more rigorously (59) than in the medical literature. Adopting this approach, it can be shown that substituting an equivalent NaCl concentration for the complex ionic composition of saliva (62) is a poor approximation that leads to overestimation of partially hydrated diameter in the most detailed published drying model (55). Saliva also contains surfactant proteins (63) which may influence the Kelvin effect and hence the equilibrium hydration of small aerosol particles.

These issues may be responsible for a quantitative discrepancy in drying behavior observed between saliva and simple aqueous media (55) although the drying curves published for cough droplets (42) and natural aerosol droplets (59) are qualitatively similar. Saliva also contains 1–2% glycoproteins and mucopolysaccharides (27), which have been considered simply as insoluble solids contributing to the size of the droplet nucleus (55). Such polymers also contribute viscosity and are known to hinder the rehydration of dried residues, at least at macroscopic length scales (64). They might therefore retard the rehydration of aerosol nuclei when the humidity rises on inhalation, allowing them to lodge deeper in the respiratory tract.

### Coalescence and Fragmentation of Droplets

Droplets are generated when surface fluid is detached and fragmented in the strong airflows of sneezing, coughing, and speech (25, 26). Fragmentation continues in the shear field of the violently expelled air (37, 65), prolonged by the viscoelasticity of the mucus polymers (66). In turbulent airflows, collisions between droplets can lead to either fragmentation or coalescence. Collisions occur when large droplets are pulled through a mist of small droplets by gravity, as in rain clouds, or by centrifugal force in turbulent eddies. Colliding droplets may fuse or may separate again, leaving a spray of smaller droplets between them (67, 68). The outcome of droplet collisions can be affected by electrostatic effects but if significant, these are hard to predict (69). There are large non-intuitive effects of surfactants (70) and viscosity (71) that might be relevant to droplets containing SARS-CoV-2.

Exhaled aerosols can also coalesce with natural water droplets (mist) or with solid or liquid pollution particulates (smoke or smog). SARS-CoV-2 sorbed on air particulates has been observed (72). There is mounting evidence for association of Covid-19 outbreaks with conditions where there are high levels of particulate pollution in the size range 0.2–10 μm (73). Such associations been observed in Italy (74–76), China (77), the USA (78), and Iran (79). How air pollution might enhance transmission of SARS-CoV-2 is not clear: effects on the respiratory physiology of recipients (80) are not excluded (76). The reactive environment of smog particles (81) does not seem likely to enhance the stability of viruses, but sorption into porous carbon (soot) particles would give protection from sunlight. The mechanism of interaction of SARS-CoV-2 with airborne particulates is a current research gap. Until more is known it would seem prudent to segregate pedestrians from traffic in places like busy city streets and around school entrances. In less developed regions, the combination of poorly ventilated housing and smoke from cooking fires may exacerbate infection hazards (82).

### Virus Stability and Inactivation in Aerosols

SARS-CoV-2 is viable with a half-life of approximately an hour in artificially generated aerosols (5, 7, 83) much shorter than on hard surfaces (6, 8, 84). A preprint suggests some residual viability up to almost a day, longer than for other coronaviruses (85). Many viruses are sensitive to temperature and humidity (86) but effects of humidity on SARS-CoV-2 in aerosols have been considered quite small (5, 7), in contrast to its effect on viability in surface residues (84). A suggestion that SARS-CoV-2 is inactivated by specific combinations of temperature and humidity needs experimental confirmation (87). Strong sunlight reduces the half-life in aerosols to 2–3 min (7). The UV component of sunlight is likely to be responsible (88). UVB and UVC do not pass through window glass. UVC radiation is in general the most effective waveband for virus inactivation (89).

### Transport of Aerosols in Moving Air

Large (>50 μm) droplets are directly infective only if they reach another person before settling below face height (46, 55). That is the idea underlying social distancing guidelines of 1 or 2 m, although violent coughing or sneezing can carry the virus >2 m (90). Aerosol particles move with the air. Remaining infective for an hour or more, they can potentially travel much greater distances in that time (10), although social distancing is still effective because the virus concentration is reduced by dispersion (91). Using published data for vertical and horizontal dispersion of a cough jet (55) and assuming similar dispersion along the jet axis, the aerosol concentration appears to fall by a factor of about 7 from 1 m distance to 2 m distance from the source, roughly in line with existing social distancing measures based on large-droplet trajectories. However, an important gap in our knowledge is how the effectiveness of dispersion depends on environmental conditions, particularly turbulence: an infectious cloud in gentle convection movement, for example, might stay compact over comparatively long distances.

In still air the plume of warm breath rises above the emitting person (10), and even the aerosol fraction projected during a cough rises slightly (55). Thus, a person standing is more exposed to aerosol infection from a person sitting, the converse of infection by larger droplets. Opening and closing doors moves aerosols from room to room (10) and a person walking tows a potentially infective wake behind them (10, 92), in which the turbulent airflow is complex with a tendency to draw downward behind the head (93). Wind obviously carries and disperses aerosols, and its turbulence may keep larger particles airborne (61). Downwind infection is therefore a hazard, for example in street cafes, but wind movements in built-up environments are complex and difficult to model. Modeling of the transport of environmental pollutants [e.g., (94, 95)] may provide a starting point.

## Practical Implications for COVID-19 Control

### Implications for Ventilation

The survival of SARS-CoV-2 in aerosol form means that ventilation can have both positive and negative impacts. In an enclosed space, the airborne viral concentration from an infected person will build up over time to a level that depends on the ratio of the emission rate (44) to the number of fresh-air exchanges per hour (23, 96). The risk then depends on the duration of exposure (16) as well as the fresh air ventilation rate. Conversely, long-distance indoor transport by natural or mechanical ventilation is a potential hazard that does not exist for infection by larger droplets (23, 97, 98).

To minimize infection, heating and ventilation in public buildings and in transportation may need to be modified or operated in different ways from those intended at installation (87). This provides opportunities for rapid, simple interventions (17, 99–102). These were noted by the building services industry at an early stage of the pandemic, and detailed practical guidance is available for an American context (101, 102) and from trade associations in Europe (103, 104) and the UK (105). Hospital ventilation is not considered here because it is designed to prevent infection (86).

The principle that air should move from clean to potentially contaminated spaces (100) is more difficult to implement when it is not known who is infected. If possible, air should not flow from any person toward other people, especially at face height. Above-seat ventilators on coaches (60) and aircraft (19, 106) may cause exactly that if used inappropriately. In public buildings, clean air may be obtained by recirculating through HEPA filters (19, 102, 107, 108) or by ventilating with outside rather than recirculated air (103, 105) or simply by opening windows (102), accepting that indoor air temperatures may then be colder than guideline limits in winter or hotter in summer. Old and repurposed buildings are particularly challenging and may need to be individually assessed for potential hazards. Portable air filtration units may have applications in these settings (107–110). Intelligently placed screens (111) may be effective in reducing exposure by disturbing the airflow. Air conditioning or heating set to recirculate may also transfer aerosols between car or taxi passengers (19).

### Implications for Virus Inactivation

Unless SARS-CoV-2 can be inactivated by changing humidity or temperature (87), UV radiation seems more promising (7, 112). It would be helpful to know more about the wavelength sensitivity of the virus (7) for insights into effects of weather (113) and of opening windows to let sunlight UV enter. Within the limitation of their direct hazard to humans, UVC lamps as used in the food industry are a promising countermeasure (88, 89), although they have not prevented COVID-19 clusters centered on meat processing plants (114). UVC radiation may have value in treatment of aerosols in unoccupied spaces such as lift shafts, ventilation ducts and beamed under high ceilings where rising aerosols collect (112, 115–117). There could be opportunities to programme UV lamps in lift compartments, stairwells and corridors to switch off when motion sensors switch lighting on.

### Implications for Mask Design

There is epidemiological evidence that masks reduce infection, even when imperfect (118, 119). Any face covering will catch large droplets from a cough or sneeze (120), but aerosol particles follow the airflow and escape through any gaps at the edges (19). Good fit is therefore important (121, 122). It might be expected that aerosols would be challenging to filter because the droplet diameter is smaller than the mesh size. However, the choice of filter materials depends on some quite complex physics including coagulation, surface adhesion (123) electrostatic interactions (124). A fairly wide range of multilayer filters (125–127) and even some combinations of natural fibers (121, 126) seem to give worthwhile filtration of aerosol-size particles in practice. Even single cloth layers that do not capture aerosols reduce the range of exhaled air (119, 122, 128), Rapid screens for filtration efficiency are available (129, 130). There is no support for the argument that aerosol transmission makes masks useless (120).

## Discussion

Airborne transmission of SARS-CoV-2 is a significant factor in the pandemic, not yet tightly quantified but possibly comparable in magnitude to the accepted transmission routes via large droplets and surface deposits. Accumulation of infective aerosols in indoor spaces where ventilation is inadequate or largely recirculated means that exposure time is a key factor (16), and helps to explain why asymptomatic individuals, including young people, participate in the transmission chain (14). Social distancing (91) and well-fitting masks (121) help to reduce aerosol transmission as well as large droplet transmission, but other precautions specific to aerosols are also needed. These could include operational changes to ventilation systems in public buildings and public transport (98–101), UV lamps in some indoor locations (112, 116, 117), and attention to wind (61) and sunlight (7) in outdoor settings.

New knowledge about SRAS-CoV-2 is desperately needed, and is accumulating fast. Some knowledge gaps identified here include the nature of “superspreader” events; experimental data on the evolution of droplet size after emission; coalescence with air pollutants; effective wavebands of UV radiation; and the dispersal of aerosols in airflows, influencing requirements for social distancing.

## Author Contributions

The author confirms being the sole contributor of this work and has approved it for publication.

## Conflict of Interest

The author declares that the research was conducted in the absence of any commercial or financial relationships that could be construed as a potential conflict of interest.
